# Case report: A new *de novo* mutation of the Troponin T2 gene in a Chinese patient with dilated cardiomyopathy

**DOI:** 10.3389/fcvm.2023.1288328

**Published:** 2023-11-20

**Authors:** Huan Yang, Ke Gong, Yong Luo, Lei Wang, Zhiping Tan, Yao Yao, Li Xie

**Affiliations:** ^1^ Department of Pulmonary and Critical Care Medicine, Hunan Provincial People’s Hospital, The First Affiliated Hospital of Hunan Normal University, Changsha, China; ^2^ Department of Cardiovascular Surgery, The Second Xiangya Hospital of Central South University, Central South University, Changsha, China; ^3^ Department of Blood Transfusion, The Second Xiangya Hospital of Central South University, Central South University, Changsha, China

**Keywords:** dilated cardiomyopathy, mutation, TNNT2, genome, whole-exome sequencing

## Abstract

Dilated cardiomyopathy (DCM) is a cardiovascular disease characterized by persistent ventricular dilatation and systolic dysfunction. DCM has a variety of causes, including myocarditis; exposure to narcotics, alcohol, or other toxins; and metabolic or endocrine disorders. Genetic factors play a dominant role in 30%–40% of DCM cases. Here, we report a case of DCM with very severe heart failure. Because of the severity of heart failure, the patient underwent heart transplantation. We speculated that the patient's DCM might be due to a mutation; hence, we performed whole-exome sequencing of the patient and their parents, which showed a *de novo* heterozygous mutation (NM_001001431.2c.769G>A:p.E257K) in *TNNT2*, which was considered pathogenic according to the ACMG pathogenicity assessment. This finding expands the genetic map of DCM and *TNNT2* and will be important for future studies on the genetic and disease relationships between DCM and *TNNT2*.

## 1. Introduction

Dilated cardiomyopathy (DCM) is a cardiovascular disease characterized by continuous ventricular dilatation and systolic dysfunction, with an incidence of approximately 1 in every 2,500 individuals (1–3). Due to its high prevalence, morbidity, and mortality, as well as frequent hospitalizations, DCM is a significant public health concern among adults. DCM has numerous causes, including myocarditis; exposure to narcotics, alcohol, or other toxins; and metabolic or endocrine disorders. In 30%–40% of DCM cases, genetic mutations involving genes responsible for cytoskeleton, sarcomere, and nuclear envelope proteins are observed (4, 5). Due to the heterogeneity of DCM etiology, a thorough diagnostic evaluation is required to identify the specific underlying cause and exclude other diseases with overlapping phenotypes (6).

Currently, over 50 genes encoding cytoskeletal, nuclear skeleton, mitochondrial, and calcium-handling proteins are linked to hereditary DCM. Pathogenic variants of DCM have been reported with a heterogeneous group of genes encoding proteins with different functions, such as sarcomere integrity and force transmission, cytoskeletal architecture, cell–cell contact, nuclear organization, transcription, and ion channel activity. Although extensive genetic heterogeneity is present, titin (TTN) is the most common disease gene accounting for 20%–25% of the genetic causes (7). The second most prevalent gene is lamin A/C (LMNA), which accounts for about 6% of the genetic causes, followed by beta-myosin heavy chain (MYH7) (8). Several other genes account for 1%–2% of familial cases, while many others are less frequent or even reported only once. Alterations, such as copy number variations (CNVs), have been infrequently reported in association with DCM (9). This disorder is therefore regarded as a complex of multiple genetic and structural disorders. Recently, whole-exome sequencing (WES) has been utilized more frequently to examine the genetic basis of the disease by sequencing the protein-coding exome. This method of genetic screening for extremely uncommon Mendelian disorders is accurate and cost-effective (6). In addition, unlike traditional sequencing methodologies, WES does not require *a priori* assumptions regarding the causes of disease (10). Troponin T (TNNT2) cardiomyopathy is an aggressive, early-onset disease accompanied by substantial morbidity and mortality. Elegant functional investigations have previously demonstrated distinctive changes in calcium sensitivity and contractility in DCM and hypertrophic cardiomyopathy (HCM) resulting from *TNNT2* mutations.

Here, we describe a rare case of *TNNT2* mutation resulting in dilated cardiomyopathy. The patient had to undergo a heart transplantation due to the severity of heart failure.

## 2. Case report

### 2.1. Clinical information

**Figure 1:**
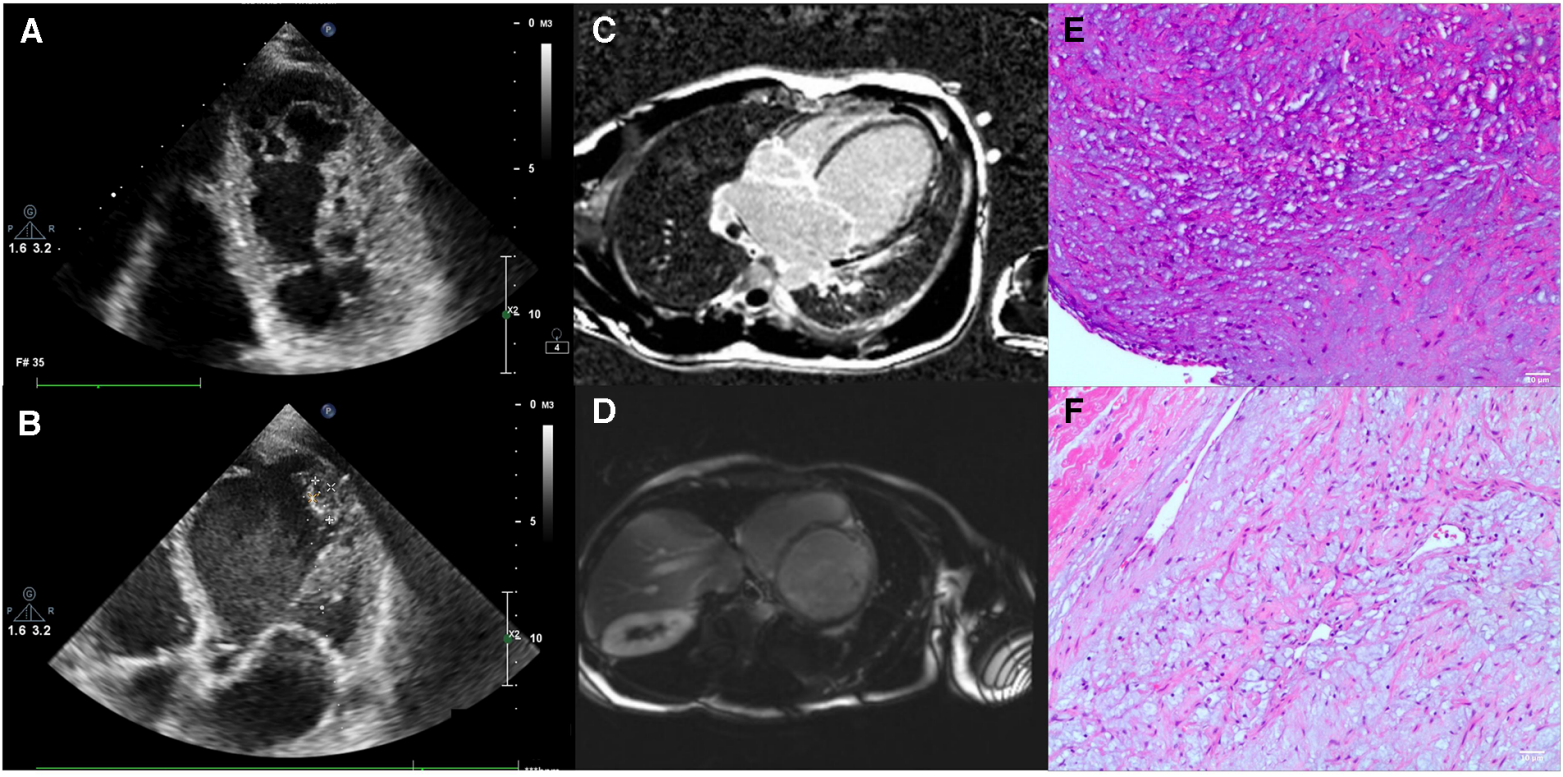
The patient's examination results. (**A,B**) Show the echocardiographic results of the patient's heart. (**C,D**) Show the images from a cardiac MRI examination. (**E,F**) show the pathological examination of recipient heart specimens from patients.

The patient was a 31-year-old man who experienced recurrent shortness of breath and poor appetite for 3 months, which worsened in the last 2 weeks. The patient was initially hospitalized, and echocardiography revealed the following values: LVED 78 mm, LAS 58 mm, RVD 39 mm, FAS 78 mm, IVsd 8 mm, LVPWd 8 mm, and EF20%. The results of the color Doppler ultrasound suggested global enlargement of the heart and generally low and uncoordinated ventricular wall motion. Myocardial tissue at the apex of the left ventricle was mildly lax. The apices of the left and right ventricles were evaluated for thrombosis. We found mild aortic regurgitation, moderate-severe bicuspid valve regurgitation, and mild tricuspid valve regurgitation. The pulmonary artery was moderately dilated and had mild regurgitation. We estimated an increase in the pulmonary artery pressure. Moreover, his left ventricular function had deteriorated (Figures 1A, B). He was diagnosed with DCM, moderate-severe bicuspid regurgitation, left ventricular apical thrombosis, right ventricular apical thrombosis, occasional ventricular premature beats and paroxysmal ventricular tachycardia, right pleural effusion, chronic heart failure, acute heart failure, and heart function grade 2163;. The patient was discharged 1 week after receiving symptomatic and supportive interventions (cardiotonic, diuresis, ventricular rate control, and inhibition of myocardial remodeling). Post-discharge, the patient received anti-heart failure medication on a regular basis. Three months later, the patient was readmitted to the hospital due to recurrent shortness of breath and poor appetite for 7 months, which had worsened over 20 days. We speculated dilated cardiomyopathy. After hospitalization, the patient was administered cardiotonic, diuretic, anti-heart failure, transplantation of myocardial remodeling, and prognostic enhancement medication. The patient continued to be treated post-discharge with cardiotonic, diuretic, and other medications, but the symptoms of chest constriction and shortness of breath persisted. The patient sought admission to our department for surgical treatment.

Cardiac magnetic resonance imaging (MRI) revealed a small amount of delayed enhancement in the left ventricular septal wall, consistent with dilated cardiomyopathy and tricuspid insufficiency (Figures 1D, E). A coronary computed tomography angiography (CTA) was also performed, and the coronary arteries were found to be unproblematic. After matching with a donor, we promptly performed a heart transplantation using the dual-chamber method. The patient was discharged from the hospital 3 weeks after surgery. Hematoxylin and eosin (HE) staining revealed focal interstitial fibrous hyperplasia with vitreous change, mucinous degeneration, angiogenesis, and dilated muscle red tissue (Figures 1E, F).

### 2.2. Genetic analysis

**Figure 2:**
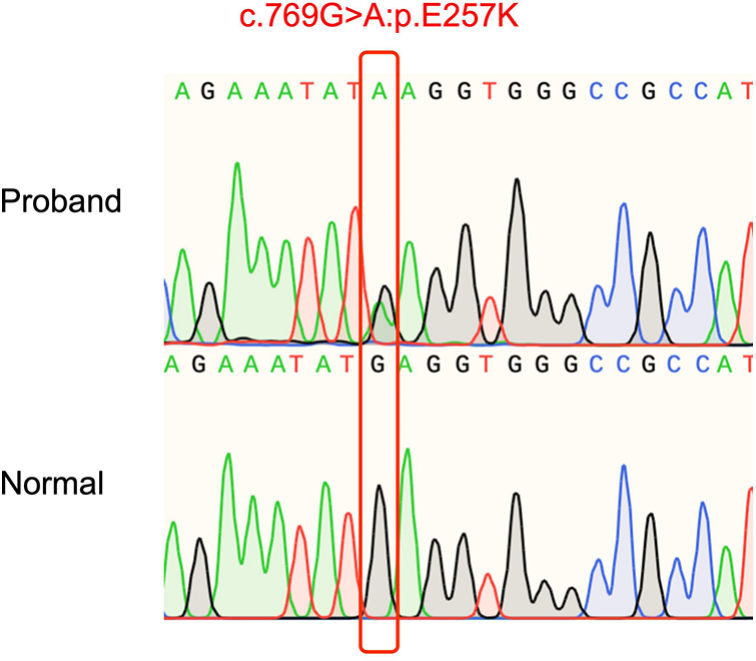
The patient's mutation site.

DNA from the patient and his parents was extracted from peripheral venous blood. Genomic DNA was prepared using the DNeasy Blood & Tissue Kit (Qiagen, Valencia, CA, USA). The majority of WES and CNV sequencing (Beijing, China) was completed by the Berry Genomics Bioinformatics Institute. Exons were extracted using the Agilent SureSelect Human All Exon V6 reagent, and high-throughput sequencing was performed using the Illumina HiSeq X-10. The Berry Genomics Bioinformatics Institute also performed fundamental bioinformatics analyses, including reads, mapping, variant detection, filtering, and annotation. We then filtered the data as follows: (1) we ranked genes using Polyphen-2, SIFT, MutationTaster, and CADD; (2) we excluded non-coding regions and synonymous variants that had no effect on splicing; and (3) we examined variants in the 1,000 g, Exome Aggregation Consortium, and gnomAD databases. Sanger sequencing was used to identify a unique non-synonymous variant. WES revealed a *de novo* heterozygous mutation *TNNT2* (NM_001001431.2c.769G>A:p.E257K) in the patient, which was absent in the patient's parents (Figure 2). Based on the ACMG pathogenicity assessment, we chose PM1 + PM2 + PM6 + PP3 + PP4 to evaluate this mutation as likely pathogenic. Consequently, this patient's DCM may have been caused by a mutation in this gene.

## 3. Discussion

DCM is a prevalent primary myocardial disease characterized by left ventricular or biventricular systolic dysfunction, which frequently leads to heart failure, arrhythmia, thromboembolism, and sudden mortality. DCM is associated with the dysfunction of diverse pathways, including Z discs, nuclear lamins, intermediate filaments, and dystrophin-associated glycoprotein complexes (11), in contrast to hypertrophic cardiomyopathy (HCM), which is strongly associated with myosin mutations. Idiopathic DCM is diagnosed when detectable causes of DCM are ruled out (12). These include hypothyroidism, chemotherapy medications, alcoholism, ischemia, and known pathogenic mutations. Studies on DCM have determined that 20% to 50% of DCM cases are due to familial inheritance (13, 14), which is associated with rare mutations in more than 40 genes (15). In 90% of cases, inheritance is autosomal dominant, while autosomal recessive and X-linked inheritance are uncommon (4). The majority of these mutations are single-nucleotide variants that alter the amino acid sequence of the encoded protein. Rare mutations consist of minor in-frame insertions or deletions, but rarely large deletions (16).

At present, the main pathogenic genes of DCM include *TTN*, *LMNA, RBM20*, and *DES*. The discovery of the role of TTN truncating variants in DCM has been a major advance (17). *TTN* encodes the giant protein titin, the largest known protein expressed in the heart. Titin acts as a spring, providing the drive for and regulating sarcomere contraction and signaling (18). Missense variants in *TTN* have a high prevalence, both rare and common (7). Missense and truncating mutations in *LMNA* account for 5%–8% of inherited DCM cases (19). As with *TTN*, *LMNA* mutations are inherited in an autosomal dominant manner. A single *LMNA* gene encodes laminin A and C, and differential splicing of the 3′ termini results in the two proteins being identical for their first 566 amino acids. *LMNA* mutations result in various diseases, from premature aging to myopathy and DCM (20, 21). RNA-binding motif 20 (RBM20) is an RNA-binding protein that is highly expressed in the atrium and ventricle. Dominant mutations in the *RBM20* gene were first described in DCM, accounting for 1%–5% of DCM cases (22). ipSC-derived cardiomyocytes with the *RBM20* R636S mutation generate gene expression and splicing profiles consistent with cardiomyopathy, affecting not only *TTN* but also *CAMK2D* and *CACNA1C* (23, 24). DES encodes the protein desmin, a functional structure in cardiac myocytes in terms of cellular mechanical stability and connectivity. Desmin functions as part of the dystrophin-associated glycoprotein complex and primarily affects the cytoskeleton (25). The DES variant is associated with hopelessness and affected patients mainly present with muscle weakness, conduction block, and DCM (26, 27).

*TNNT2* (OMIM: 191045) encodes the thin filament contractile protein cardiac troponin T, a troponin complex that binds to tropomyosin in sarcomeres (28). The 25 kb genome of *TNNT2* contains 16 exons and is located on chromosome 1q32. Cardiac troponin (cTn) consists of three distinct subunits, each of which is named for its function: cardiac troponin I (cTnI) inhibits actin ATPase activity independently of other Tn subunits; cardiac troponin C (cTnC) binds Ca^2+^ to low-affinity Ca^2+^-specific binding sites to alleviate cTnI inhibition (29, 30); and cardiac troponin T (cTnT) attaches tropomyosin (Tm) to the entire cTn complex. Recent investigations have revealed that cardiac TnT is essential not only for the structural integrity of the troponin complex but also for sarcomere assembly and cardiac contractility (31). The troponin complex is a calcium sensor that regulates striated muscle contraction and modifies the activity and force of actin ATPase (32). *TNNT2* gene mutations have been linked to familial HCM and DCM over the past decade (33–35). Numerous studies on recombination systems have provided valuable information on the functional impact of TnT disease-related mutations (36, 37).

We described a DCM patient with a mutated *TNNT2* gene. DCM was confirmed following a comprehensive examination of this patient. WES was then conducted, and sequencing revealed a *de novo* mutation that had not been previously reported. According to the ACMG pathogenicity assessment, we determined that this mutation was likely pathogenic. This patient most likely experienced a condition associated with this mutation. According to reports, truncating troponin T may result in a significant decrease in force production during cardiac contraction (38). It is possible that troponin T deficiency stimulates cardiac enlargement by increasing myocardial power and energy burden and decreasing force production during cardiac contraction. In addition, cTnT, composed of troponin C, troponin I, and troponin T, regulates myocardial contraction and relaxation by controlling calcium concentration-dependent interactions of 2+ actin (39). *TNNT2* gene mutations may affect the complex stability and/or interaction between myosin and troponin T, which may alter the actin interaction. Furthermore, *TNNT2* gene mutations can reduce the sensitivity of the complex to Ca^2+^, thereby reducing the contractility of the myocardium. In contrast, mutations in genes encoding sarcomere proteins associated with HCM can increase Ca^2+^ sensitivity, thereby increasing cardiac contractility (40). Thus, mutations in different proteins may be associated with common cardiac phenotypes, DCM or HCM, depending on the ultimate effect of the mutation on the strength of myocardial contraction. The mutation in *TNNT2* results in a disease with an early onset and a poor prognosis. Therefore, DCM patients with a *TNNT2* mutation should receive early intervention, routine follow-ups, and surgical treatment for heart failure.

In summary, we report a novel mutation in *TNNT2* in a patient with DCM, which expands the genetic map of DCM and *TNNT2*.

## Data Availability

The data presented in the study are deposited in the dbSNP repository, accession number rs1131691898.

## References

[1] Reichart D Magnussen C Zeller T Blankenberg S. Dilated cardiomyopathy: from epidemiologic to genetic phenotypes: a translational review of current literature. J Intern Med. (2019) 286(4):362–72. 10.1111/joim.12944 31132311

[2] Taylor MR Carniel E Mestroni L. Cardiomyopathy, familial dilated. Orphanet J Rare Dis. (2006) 1:27. 10.1186/1750-1172-1-27 16839424 PMC1559590

[3] Tharp CA Haywood ME Sbaizero O Taylor MRG Mestroni L. The giant protein titin’s role in cardiomyopathy: genetic, transcriptional, and post-translational modifications of TTN and their contribution to cardiac disease. Front Physiol. (2019) 10:1436. 10.3389/fphys.2019.01436 31849696 PMC6892752

[4] Hershberger RE Morales A Siegfried JD. Clinical and genetic issues in dilated cardiomyopathy: a review for genetics professionals. Genet Med. (2010) 12(11):655–67. 10.1097/GIM.0b013e3181f2481f 20864896 PMC3118426

[5] Merlo M Cannata A Gobbo M Stolfo D Elliott PM Sinagra G. Evolving concepts in dilated cardiomyopathy. Eur J Heart Fail. (2018) 20(2):228–39. 10.1002/ejhf.1103 29271570

[6] Duboscq-Bidot L Xu P Charron P Neyroud N Dilanian G Millaire A Mutations in the Z-band protein myopalladin gene and idiopathic dilated cardiomyopathy. Cardiovasc Res. (2008) 77(1):118–25. 10.1093/cvr/cvm015 18006477

[7] Gerull B Gramlich M Atherton J McNabb M Trombitas K Sasse-Klaassen S Mutations of TTN, encoding the giant muscle filament titin, cause familial dilated cardiomyopathy. Nat Genet. (2002) 30(2):201–4. 10.1038/ng815 11788824

[8] Fatkin D MacRae C Sasaki T Wolff MR Porcu M Frenneaux M Missense mutations in the rod domain of the lamin A/C gene as causes of dilated cardiomyopathy and conduction-system disease. N Engl J Med. (1999) 341(23):1715–24. 10.1056/NEJM199912023412302 10580070

[9] Gerull B Klaassen S Brodehl A. The genetic landscape of cardiomyopathies. Genet Causes Cardiac Disease. (2019):45–91. 10.1007/978-3-030-27371-2_2

[10] Elliott PM Anastasakis A Borger MA Borggrefe M Cecchi F Charron P 2014 ESC guidelines on diagnosis and management of hypertrophic cardiomyopathy: the task force for the diagnosis and management of hypertrophic cardiomyopathy of the European society of cardiology (ESC). Eur Heart J. (2014) 35(39):2733–79. 10.1093/eurheartj/ehu284 25173338

[11] Dellefave L McNally EM. The genetics of dilated cardiomyopathy. Curr Opin Cardiol. (2010) 25(3):198–204. 10.1097/HCO.0b013e328337ba52 20186049 PMC2939233

[12] Hirtle-Lewis M Desbiens K Ruel I Rudzicz N Genest J Engert JC The genetics of dilated cardiomyopathy: a prioritized candidate gene study of LMNA, TNNT2, TCAP, and PLN. Clin Cardiol. (2013) 36(10):628–33. 10.1002/clc.22193 24037902 PMC6649360

[13] Burkett EL Hershberger RE. Clinical and genetic issues in familial dilated cardiomyopathy. J Am Coll Cardiol. (2005) 45(7):969–81. 10.1016/j.jacc.2004.11.066 15808750

[14] Zhang XL Qiu XB Yuan F Wang J Zhao CM Li RG TBX5 loss-of-function mutation contributes to familial dilated cardiomyopathy. Biochem Biophys Res Commun. (2015) 459(1):166–71. 10.1016/j.bbrc.2015.02.094 25725155

[15] Fatkin D Otway R Richmond Z. Genetics of dilated cardiomyopathy. Heart Fail Clin. (2010) 6(2):129–40. 10.1016/j.hfc.2009.11.003 20347783

[16] Fokstuen S Lyle R Munoz A Gehrig C Lerch R Perrot A A DNA resequencing array for pathogenic mutation detection in hypertrophic cardiomyopathy. Hum Mutat. (2008) 29(6):879–85. 10.1002/humu.20749 18409188

[17] Herman DS Lam L Taylor MR Wang L Teekakirikul P Christodoulou D Truncations of titin causing dilated cardiomyopathy. N Engl J Med. (2012) 366(7):619–28. 10.1056/NEJMoa1110186 22335739 PMC3660031

[18] LeWinter MM Granzier HL. Cardiac titin and heart disease. J Cardiovasc Pharmacol. (2014) 63(3):207–12. 10.1097/FJC.0000000000000007 24072177 PMC4268868

[19] van Tintelen JP Hofstra RM Katerberg H Rossenbacker T Wiesfeld AC du Marchie Sarvaas GJ High yield of LMNA mutations in patients with dilated cardiomyopathy and/or conduction disease referred to cardiogenetics outpatient clinics. Am Heart J. (2007) 154(6):1130–9. 10.1016/j.ahj.2007.07.038 18035086

[20] Lu JT Muchir A Nagy PL Worman HJ. LMNA cardiomyopathy: cell biology and genetics meet clinical medicine. Dis Model Mech. (2011) 4(5):562–8. 10.1242/dmm.006346 21810905 PMC3180218

[21] Keller H Finsterer J Steger C Wexberg P Gatterer E Khazen C Novel c.367_369del LMNA mutation manifesting as severe arrhythmias, dilated cardiomyopathy, and myopathy. Heart Lung. (2012) 41(4):382–6. 10.1016/j.hrtlng.2011.07.007 22019351

[22] Brauch KM Karst ML Herron KJ de Andrade M Pellikka PA Rodeheffer RJ Mutations in ribonucleic acid binding protein gene cause familial dilated cardiomyopathy. J Am Coll Cardiol. (2009) 54(10):930–41. 10.1016/j.jacc.2009.05.038 19712804 PMC2782634

[23] Wyles SP Li X Hrstka SC Reyes S Oommen S Beraldi R Modeling structural and functional deficiencies of RBM20 familial dilated cardiomyopathy using human induced pluripotent stem cells. Hum Mol Genet. (2016) 25(2):254–65. 10.1093/hmg/ddv468 26604136 PMC4706113

[24] Gaertner A Klauke B Felski E Kassner A Brodehl A Gerdes D Cardiomyopathy-associated mutations in the RS domain affect nuclear localization of RBM20. Hum Mutat. (2020) 41(11):1931–43. 10.1002/humu.24096 32840935

[25] Hershberger RE Siegfried JD. Update 2011: clinical and genetic issues in familial dilated cardiomyopathy. J Am Coll Cardiol. (2011) 57(16):1641–9. 10.1016/j.jacc.2011.01.015 21492761 PMC3088091

[26] McNally EM Golbus JR Puckelwartz MJ. Genetic mutations and mechanisms in dilated cardiomyopathy. J Clin Invest. (2013) 123(1):19–26. 10.1172/JCI62862 23281406 PMC3533274

[27] Brodehl A Dieding M Biere N Unger A Klauke B Walhorn V Functional characterization of the novel DES mutation p.L136P associated with dilated cardiomyopathy reveals a dominant filament assembly defect. J Mol Cell Cardiol. (2016) 91:207–14. 10.1016/j.yjmcc.2015.12.015 26724190

[28] Garcia-Castro M Reguero JR Batalla A Diaz-Molina B Gonzalez P Alvarez V Hypertrophic cardiomyopathy: low frequency of mutations in the beta-myosin heavy chain (MYH7) and cardiac troponin T (TNNT2) genes among spanish patients. Clin Chem. (2003) 49(8):1279–85. 10.1373/49.8.1279 12881443

[29] Solaro RJ Rarick HM. Troponin and tropomyosin: proteins that switch on and tune in the activity of cardiac myofilaments. Circ Res. (1998) 83(5):471–80. 10.1161/01.res.83.5.471 9734469

[30] Farah CS Reinach FC. The troponin complex and regulation of muscle contraction. FASEB J. (1995) 9(9):755–67. 10.1096/fasebj.9.9.7601340 7601340

[31] Sehnert AJ Huq A Weinstein BM Walker C Fishman M Stainier DY. Cardiac troponin T is essential in sarcomere assembly and cardiac contractility. Nat Genet. (2002) 31(1):106–10. 10.1038/ng875 11967535

[32] Potter JD Sheng Z Pan BS Zhao J. A direct regulatory role for troponin T and a dual role for troponin C in the Ca^2+^ regulation of muscle contraction. J Biol Chem. (1995) 270(6):2557–62. 10.1074/jbc.270.6.2557 7852318

[33] Kamisago M Sharma SD DePalma SR Solomon S Sharma P McDonough B Mutations in sarcomere protein genes as a cause of dilated cardiomyopathy. N Engl J Med. (2000) 343(23):1688–96. 10.1056/NEJM200012073432304 11106718

[34] Mogensen J Kubo T Duque M Uribe W Shaw A Murphy R Idiopathic restrictive cardiomyopathy is part of the clinical expression of cardiac troponin I mutations. J Clin Invest. (2003) 111(2):209–16. 10.1172/JCI16336 12531876 PMC151864

[35] Townsend PJ Farza H MacGeoch C Spurr NK Wade R Gahlmann R Human cardiac troponin T: identification of fetal isoforms and assignment of the TNNT2 locus to chromosome 1q. Genomics. (1994) 21(2):311–6. 10.1006/geno.1994.1271 8088824

[36] Szczesna D Zhang R Zhao J Jones M Guzman G Potter JD. Altered regulation of cardiac muscle contraction by troponin T mutations that cause familial hypertrophic cardiomyopathy. J Biol Chem. (2000) 275(1):624–30. 10.1074/jbc.275.1.624 10617660

[37] Venkatraman G Harada K Gomes AV Kerrick WG Potter JD. Different functional properties of troponin T mutants that cause dilated cardiomyopathy. J Biol Chem. (2003) 278(43):41670–6. 10.1074/jbc.M302148200 12923187

[38] Watkins H Seidman CE Seidman JG Feng HS Sweeney HL. Expression and functional assessment of a truncated cardiac troponin T that causes hypertrophic cardiomyopathy. Evidence for a dominant negative action. J Clin Invest. (1996) 98(11):2456–61. 10.1172/JCI119063 8958207 PMC507702

[39] Villard E Perret C Gary F Proust C Dilanian G Hengstenberg C A genome-wide association study identifies two loci associated with heart failure due to dilated cardiomyopathy. Eur Heart J. (2011) 32(9):1065–76. 10.1093/eurheartj/ehr105 21459883 PMC3086901

[40] Mirza M Marston S Willott R Ashley C Mogensen J McKenna W Dilated cardiomyopathy mutations in three thin filament regulatory proteins result in a common functional phenotype. J Biol Chem. (2005) 280(31):28498–506. 10.1074/jbc.M412281200 15923195

